# Insight into the Structural Determinants of Imidazole Scaffold-Based Derivatives as TNF-α Release Inhibitors by *in Silico* Explorations

**DOI:** 10.3390/ijms160920118

**Published:** 2015-08-25

**Authors:** Yuan Wang, Mingwei Wu, Chunzhi Ai, Yonghua Wang

**Affiliations:** 1 Lab of Systems Pharmacology, College of Life Sciences, Northwest A&F (Agriculture and Forestry) University, Yangling 712100, China; E-Mails: yuan-w-09@nwsuaf.edu.cn (Y.W.); audi.lg@163.com (M.W.); 2 Lab of Pharmaceutical Resource Discovery, Dalian Institute of Chemical Physics, Graduate School of the Chinese Academy of Sciences, Dalian 116023, China; E-Mail: yling@dicp.ac.cn

**Keywords:** imidazoles, TNF-α, inhibitor, 3D-QSAR, CoMFA, CoMSIA, DISCOtech, pharmacophore

## Abstract

Presently, 151 widely-diverse pyridinylimidazole-based compounds that show inhibitory activities at the TNF-α release were investigated. By using the distance comparison technique (DISCOtech), comparative molecular field analysis (CoMFA), and comparative molecular similarity index analysis (CoMSIA) methods, the pharmacophore models and the three-dimensional quantitative structure-activity relationships (3D-QSAR) of the compounds were explored. The proposed pharmacophore model, including two hydrophobic sites, two aromatic centers, two H-bond donor atoms, two H-bond acceptor atoms, and two H-bond donor sites characterizes the necessary structural features of TNF-α release inhibitors. Both the resultant CoMFA and CoMSIA models exhibited satisfactory predictability (with *Q*^2^ (cross-validated correlation coefficient) = 0.557, *R*^2^_ncv_ (non-cross-validated correlation coefficient) = 0.740, *R*^2^_pre_ (predicted correlation coefficient) = 0.749 and *Q*^2^ = 0.598, *R*^2^_ncv_ = 0.767, *R*^2^_pre_ = 0.860, respectively). Good consistency was observed between the 3D-QSAR models and the pharmacophore model that the hydrophobic interaction and hydrogen bonds play crucial roles in the mechanism of actions. The corresponding contour maps generated by these models provide more diverse information about the key intermolecular interactions of inhibitors with the surrounding environment. All these models have extended the understanding of imidazole-based compounds in the structure-activity relationship, and are useful for rational design and screening of novel 2-thioimidazole-based TNF-α release inhibitors.

## 1. Introduction

Chronic inflammatory diseases, such as rheumatoid arthritis (RA) and psoriatic arthritis, are highly debilitating sicknesses affecting a large segment of the population. Recently, it has become obvious that even metabolic diseases (such as type II diabetes) and cardiovascular disease (such as atherosclerosis) should also be considered to be inflammatory in nature [1]. Thus, it is not surprising that huge efforts are constantly focused on the development of anti-inflammatory drugs. The progress of chronic inflammation is driven by an amplified systemic occurrence of several proinflammatory cytokines, like tumor necrosis factor-α (TNF-α) and interleukin-1β (IL-1β), which are produced in response to infection and other cellular stresses [2]. Although an appropriate amount of TNF-α plays an important role in the host immune response, excess levels are thought to underlie a number of serious inflammatory diseases [3,4]. Recent clinical data, obtained with either chimeric TNF-α antibodies or soluble TNF-α receptor in the treatment of rheumatoid arthritis and Crohn’s disease, have confirmed the key role of TNF-α in these inflammatory disorders [5,6,7,8,9]. Antagonism of these proinflammatory cytokines has been recognized as an effective possibility for the treatment of inflammatory conditions [10].

Though the complete biochemical pathway for the production of TNF-α and IL-1β in response to inflammatory stimuli has not been elucidated yet, p38 mitogen-activated protein kinase (p38, p38 MAPK) has been demonstrated to play a central role in the inflammatory process [1,11]. P38 MAP kinases, first identified and cloned by Han *et al.* in 1994 as an important moiety of the immune response machinery activated by cytokines [12,13], were triggered by multiple extracellular stimuli, including stress signals, such as lipopolysaccharide (LPS), osmotic or heat shock, and proinflammatory cytokines, such as TNF-α or IL-1β [14,15]. Research results have shown that after the activation of p38 MAPK, pro-inflammatory cytokines, such as TNF-α and IL-1β, raised in both production and release [15]. Inversely, p38 can also be phosphorylated upon TNF-α or IL-1β receptor binding and functions in the cell signaling network responsible for the up-regulation of these inflammatory mediators, both at the transcriptional and translational level, forming a vicious cycle in the development of inflammatory response [16,17]. Importantly, previous work has demonstrated that several small-molecular inhibitors of p38 MAP kinase, which compete with ATP for access to the catalytic site, have been shown to effectively block the activation of the pro-inflammatory transcription factor AP-1, which inhibits the transcriptional induction of TNF-α, IL-1β and other cytokines and then blocks the production and release of these pro-inflammatory cytokines [1,2,11]. Thus, the biological importance of p38 MAP kinase, related to the release of pro-inflammatory cytokines, has aroused many studies aiming at the development of selective inhibitors of p38 MAP kinase for the treatment of inflammatory conditions resulting from excess cytokine production.

Most of these small-molecular inhibitors of p38 MAP kinase are derived from the prototypical pyridin-4-ylimidazole SB203580, which definitely contributes to the identification and characterization of the p38 signaling pathway as a valid therapeutic target in inflammatory conditions [1,2,11]. Presently, based on the pyridin-4-ylimidazole SB203580, Laufer *et al.* [2,10,18,19] have reported newer pyridinylimidazole inhibitors, which possess not only high p38α inhibitory activity, but also excellent inhibitory potency in suppression of the TNF-α release. In addition, these inhibitors have many other decisive advantages over prototype SB203580-like 2-arylimidazoles, e.g., higher selectivity, better kinetic and metabolic properties, and fewer interactions with metabolic enzymes like CYP450. Compared with the prototype SB203580, there are two major improvements in these newly-synthesized imidazole inhibitors: firstly, introduce additional substituents at the ortho position of the pyridin-4-yl moiety, which is sterically demanding or electronically shielding, and secondly, incorporate different substituents on the imidazole core, particularly the substituents at N1 and C2 positions of the imidazole, which might lead to improved physicochemical properties and reduced toxicity.

During the past decades, numerous new p38 inhibitors have been synthesized and reported, and to explore the structure-activity relationships of the p38 inhibitors, several groups have done excellent work on the imidazole or pyridinylimidazole inhibitors [20,21,22,23,24,25,26]. However, to our best knowledge, almost no *in silico* studies on those imidazoles inhibitors involving the inhibitory activity of TNF-α release in the human whole blood model has been reported until now. As we know, in recent years 3D-QSAR methods, like comparative molecular field analysis (CoMFA) [27] and comparative molecular similarity indices analysis (CoMSIA) [28], have been increasingly employed in rational drug discovery processes to understand the drug-receptor interaction and to design new molecules [20], due to their outstanding advantages of time-saving, cost-reducing, as well as high efficiency *in silico* screening and prediction of candidate drugs [29,30,31].

In the present work, 151 newly-synthesized imidazoles derivatives reported [2,10,18,19] as potent and selective TNF-α release inhibitors were employed as a data set to carry out a series of QSAR studies using a combination of CoMFA, CoMSIA, and pharmacophore modeling computational methods. The obtained CoMFA and CoMSIA studies not only illustrate the conformation or spatial orientation of those imidazole derivatives, but also provide useful indicators for the design of new drug candidates for inflammation diseases. These results are applicable to the prediction of the activities of new TNF-α release inhibitors and would be of help in providing structural implications for designing potent and selective TNF-α release inhibitors. Furthermore, the pharmacophore model was established to understand the essential features required for p38 binding using DISCOtech, which could provide important information for understanding of the mechanism of p38 enzyme catalysis. Based on the molecular field information of 3D-QSAR tools and pharmacophore modeling protocols, a few strategies were proposed to design new molecules with improved activities.

## 2. Results and Discussion

### 2.1. Split the Training and Test Sets

In order to validate the predicting ability of the produced 3D-QSAR models, an available dataset should be split into the training and test sets [32]. For the prediction statistics to be reliable, the selection of training and test sets should satisfy the following three rules [33,34]: (1) at least five molecules must be included in the test set; (2) the whole molecule-points of the test set in the descriptor space should be close to those in the training set; and (3) the points of the training set should be distributed evenly in the whole space possessed by the available dataset. In the present work, the Kohonen’s self-organizing maps (SOM) was employed to divide the dataset. SOM, first developed by Kohonen in the 1980s, is a class of unsupervised neural networks widely employed in classification and similarity perception [35,36]. It is a remarkable tool in exploring phase of data mining and has a special advantage of calculating various features with spatial organization to represent input signals and abstractions [37]. SOM generates a set of prototype vectors to represent the dataset and then maps these prototypes from the high-dimensional [37,38]. Taking advantage of clustering capability, SOM makes it possible that training and test sets are homogeneously distributed in the whole descriptor space and the representative molecules in both sets can describe the depth of distribution of the whole molecules [39].

**Figure 1:**
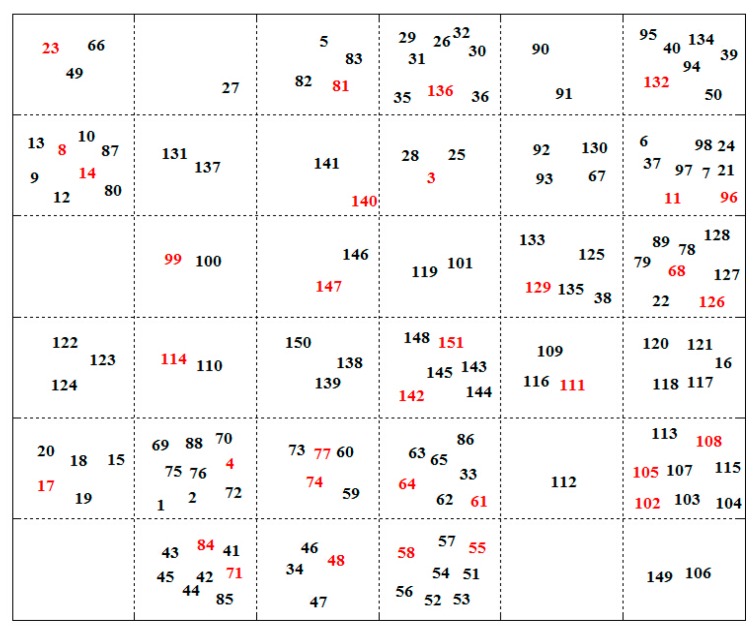
Self-organizing map showing the distribution of the training and test sets. The test set is labeled in red and the training set in black, respectively. The number equals to the series number of the molecules of the TNF-α release inhibitors.

In the present work, a total of 1664 molecular descriptors were calculated for each TNF-α release inhibitor by Dragon (version 5.4). Then, based on these descriptors as input vectors, a SOM with 6 × 6 neurons was generated for the dataset. Figure 1 shows the SOM for TNF-α release inhibitors, in which the test set is labeled in red and the training set in black, respectively. It is clear that the entire distribution of the molecules in the map is satisfactory and both sets present a uniform spread in the whole chemical space. Furthermore, the representative points in the test set are close to those in the training set. The above results indicate that the division of the dataset is reliable and rational.

### 2.2. 3D-QSAR Statistical Results

**Figure 2:**
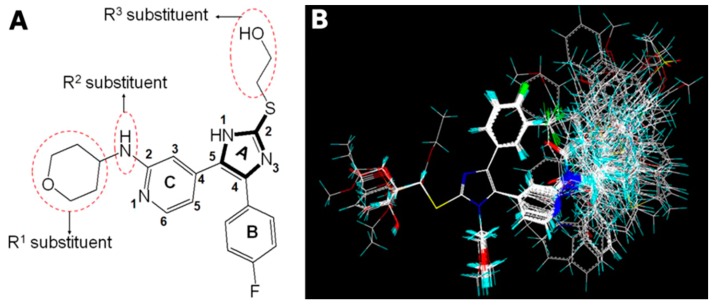
Molecular alignment of all compounds in the data set. (**A**) Compound 3v was used as a template for alignment, with the common substructure shown in bold, and three important substituents including the R^1^, R^2^, and R^3^ substituents shown in the dashed red border; and (**B**) Ligand-based alignment model of all the compounds. Molecules are colored in white for common C, blue for N, red for O, yellow for S, cyan for H, and green for F, respectively.

In the present work, the ligand-based alignment was employed to overlay the whole 151 compounds. All subsequent CoMFA and CoMSIA models were derived using the same training (118 molecules) and test (33 molecules) sets. In the present study, the most potent compound 3v was chosen as a template to fit the remaining compounds on the common substructure (Figure 2A, shown in bold) by using the “align database” command in Sybyl 6.9. Figure 2A also shows three important substituents of compound 3v, including the R^1^, R^2^, and R^3^ substituents. Figure 2B shows the resulting alignment model.

To determine the reliability of these models, all crucial statistical parameters were analyzed here, including the cross-validated correlation coefficient (*Q*^2^), non-cross-validated correlation coefficient (*R*^2^_ncv_), standard error of estimate (*SEE*), F-statistic values, the optimum number of components (*OPN*), as well as the predicted correlation coefficient (*R*^2^_pre_). *ClogP* (calculated logarithm) and hydrophobic fields are both important parameters describing the hydrophobic property of a molecule and the *ClogP* parameter always plays a crucial role in building appropriate 3D-QSAR models, which has been confirmed by many earlier studies [26,40,41,42,43]. In all of these studies, the inclusion of *ClogP* as an additional descriptor added to the QSAR models led to significantly improved statistical results of the *in silico* models. Therefore, in the present study, a 3D-QSAR analysis including *ClogP* as an additional descriptor has been carried out. Table 1 summarizes the statistical results of the CoMFA and CoMSIA analyses.

**Table 1:** Summary of CoMFA and CoMSIA results.

PLS Statistics	Model A		Model B	
	CoMFA	CoMSIA	CoMFA	CoMSIA
*Q*^2^	0.524	0.593	0.557	0.598
*R* ^2^_ncv_	0.856	0.778	0.740	0.767
*SEE*	0.323	0.399	0.432	0.409
*F*	133.655	99.117	80.388	93.091
*R*^2^_pre_	0.730	0.876	0.748	0.860
*SEP*	0.588	0.541	0.564	0.538
*OPN*	5	4	4	4
**Contribution**				
*S*	0.574	0.121	0.492	0.135
*E*	0.426	0.294	0.373	0.279
*H*	-	0.195	-	0.158
*D*	-	0.208	-	0.195
*A*	-	0.181	-	0.146
*ClogP*	-	-	0.135	0.086

*Q*^2^, cross-validated correlation coefficient; *R*^2^_ncv_, non-cross-validated correlation coefficient; *SEE*, standard error of estimate; *F*, *F*-test value; *R*^2^_pre_, predicted correlation coefficient; *SEP*, smallest predicted error; *OPN*, optimum number of components; *S*, steric hindrance; *E*, electric charge; *H*, hydrogen bond; *D*, hydrogen-bond donor; *A*, hydrogen bond acceptor; *ClogP*, calculated logarithm.

#### 2.2.1. CoMFA Details

For CoMFA analysis, the steric, electrostatic, and *ClogP* field descriptors were fitted together in every possible form to build appropriate CoMFA mathematical models. Finally, the optimal model (model A), employing both the steric and electrostatic field descriptors, obtains a LOO cross-validated *Q*^2^ of 0.524 with five components, indicating a proper internal predictive capacity of the model. A high correlation coefficient (*R*^2^_ncv_) of 0.856 for the final non-cross-validated model shows self-consistency. In addition, other statistical results including *SEE* value of 0.323, and an *F*-test value of 133.655 all suggest that the CoMFA model is a reliable predictor. While a more statistically significant model (model B) was obtained, including *ClogP* as a third parameter, which has a higher cross-validated *Q*^2^ of 0.557 with four optimum components, a non-cross-validated *R*^2^_ncv_ of 0.740, a standard error of estimation of 0.432, and *F* ratio of 80.388, proving the reliability of the model. In terms of the relative contributions in model A, the steric and electrostatic fields account for 0.574 and 0.426, respectively. In model B, the steric and electrostatic fields contribute 0.492 and 0.373, respectively, while the *ClogP* field contributes 0.135, indicating that the steric property contributes a majority to the antagonist activity.

#### 2.2.2. CoMSIA Details

Combined with *ClogP*, a total of six parameters (steric, electrostatic, hydrophobic, H-Bond donor, H–Bond acceptor, and *ClogP*) were fitted together in every possible form to build appropriate CoMSIA models. Using the same training set as in the CoMFA method, two superior models, out of all the CoMSIA models established, were obtained with high *Q*^2^ values using all the five fields (steric, electrostatic, hydrophobic, H-bond donor, and H-Bond acceptor field) (Table 1). The optimal CoMSIA model (model B), employing the *ClogP* descriptor in statistical results, showed *Q*^2^ = 0.598, *R*^2^_ncv_ = 0.767, *SEE* = 0.409, and *F* = 93.091 with four optimum components. However, another superior model without a *ClogP* index (model A) has a *Q*^2^ value of 0.593 with four optimum components, an *R*^2^_ncv_ value of 0.778, a *SEE* value of 0.399, and an *F* value of 99.117. The optimal CoMSIA model reveals that steric, electrostatic, hydrophobic, hydrogen bond-donor, and hydrogen bond-acceptor fields all have major influences in the inhibitory activity of imidazole derivatives. The corresponding field contributions of steric, electrostatic, hydrophobic, hydrogen bond-donor, and hydrogen bond-acceptor fields in model A were 0.121, 0.294, 0.195, 0.208, and 0.181, respectively, and in model B were 0.135, 0.279, 0.158, 0.195, and 0.146, respectively. Once again, the steric and electrostatic fields are demonstrated to contribute a lot to model B (with a sum of about 0.414 contribution). However, at this time, the hydrogen bond (H-Bond) descriptors, especially the H-bond fields, outstand their roles for correlation with the inhibitory activity, where the donor field possesses about 0.195 and the acceptor field occupies about 0.146 of relative contributions. Moreover, the contribution of the hydrophobic field is found to be about 0.158, while *ClogP* demonstrates a slight contribution of 0.086, suggesting a good correlation between the hydrophobic descriptor and their TNF-α release inhibitory activities. All of these conclusions can be verified in other studies on the p38 MAPK complexes that the effect of hydrogen bonding, steric and hydrophobic fields could be important for the inhibitory activity of imidazole and quinazoline functionalized inhibitors [21,26,44].

According to the above analysis, the predictive ability of both 3D-QSAR models for A are not good enough when compared with that of model B. Hence, the models including the *ClogP* parameter were selected as the optimal models and were utilized for further discussion.

#### 2.2.3. Validation of the 3D QSAR Models

To test the predictive ability of the models, the most appropriate method is to predict the activities of molecules excluded from the training set. For this purpose, the test set (33 molecules) which accounts for 28% of the training set, was used here to validate the accuracy of models A and B. We should identify possible outliers first before the final validation by the test set. Outliers from a QSAR are compounds that do not fit the model or are poorly predicted [45]. QSAR models that have very few or no outliers are known as good models. On the other hand, those QSAR models that have a large number of outliers are bad models. Many reasons may account for the presence of outliers in the dataset used for *in silico* modeling, including unique structural differences, different binding conformation, or a higher residual between the observed and predicted biological activity of an inhibitor [46,47].

However, the removal of outliers will be allowed for the development of stronger and more significant models and the outlier test is, therefore, reasonable and necessary in the derived models. There are a variety of methods to highlight outliers, including identifying those compounds with significantly high residuals from regression-based techniques. At present, the proposed models (CoMFA and CoMSIA) are checked to identify possible outliers. It can be observed that no residuals in both training and test sets are more than 1.5 log units, illustrating that the CoMFA and CoMSIA models are both robust and predictive. Thus, it is reasonable to consider that there are no outliers in the present models.

**Figure 3:**
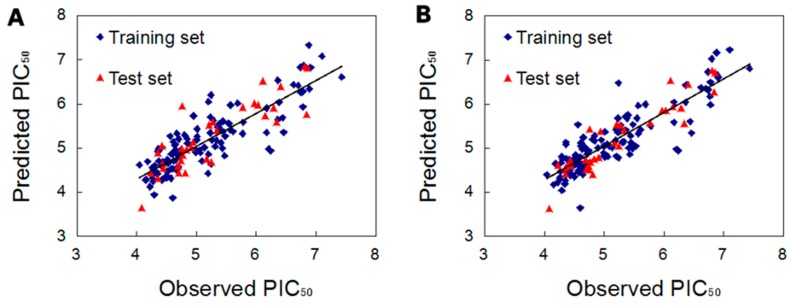
The correlation plots of the predicted pIC_50_ values *versus* the observed pIC_50_ values using the training set and the test set based on (**A**) CoMFA model and (**B**) CoMSIA model. The solid lines are the regression lines for the fitted and predicted bioactivities of training and test compounds, respectively. (more detail in Table S1).

After outlier testing, both CoMFA and CoMSIA models in model B exhibit good prediction ability, yielding *R*^2^_pre_ of 0.748 for CoMFA and 0.860 for CoMSIA, respectively. The plot of actual *versus* predicted activities of all compounds for the optimal CoMFA and CoMSIA models is depicted in Figure 3A,B, respectively, where the data points are rather uniformly distributed around the regression line, indicating the reasonability of the obtained models.

### 2.3. Interpretation of 3D-QSAR Contour Maps

**Figure 4:**
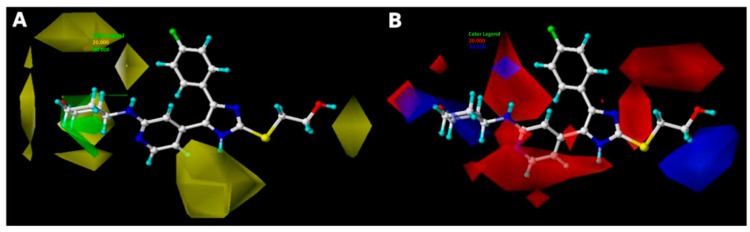
CoMFA StDev*Coeff contour plots. (**A**) Steric (green/yellow) contour map in combination with compound 3v in ball and stick. Green contours indicate regions where bulky groups increase the activity; yellow contours indicate regions where bulky groups decrease the activity; and (**B**) Electrostatic contour map (red/blue) in combination with compound 3v. Red contours indicate regions where negative charges increase the activity; blue contours indicate regions where positive charges increase the activity.

After consideration of both the internal and external predictive powers of all the derived models, the best CoMFA and CoMSIA models are selected to construct the standard deviation × coefficient (StDev*Coeff) contour maps to view the field effects on the target features. All contour maps obtained from the models are illustrated together with the most potent compound 3v (Figure 4A). In order to select appropriate contour levels for each feature, the resulting histograms of actual field values were analyzed, and a contour level was chosen interactively as those that produce the best interpretable contour plot.

Figure 4A depicts the CoMFA steric contour map of the optimal model with compound 3v overlaid. In this map, the green (sterically-favorable) and yellow (sterically-unfavorable) contours represent 80% and 20% level contributions, respectively. The green contour region around ring C (R^1^ substituent) indicates that a bulky substituent is preferred in this position to produce higher inhibitory activity. Compounds with high potency such as 30h, 3v, and 3w have sterically-favoured groups at the R^1^ position occupying the big green contour. However, due to the absence of such groups in the sterically-favoured green areas, compounds 5k, 5i, and 5f show much lower activity. There are large yellow contours near the green contour region, which suggests that substitution of too bulky a group at R^1^ substituent would lead to a decrease in activity, such as compounds 22j and 46 (with C_11_H_16_ and C_14_H_20_O at the R^1^ substituent position, respectively). In addition, a medium-sized yellow contour is seen in the vicinity of the OH (R^3^ substituent), which suggests that occupancy of this sterically-unfavorable region with bulky substituent would have a detrimental effect on the inhibitory activity. This discovery is well illustrated by the example that compound 1b with –CH(COOCH_2_CH_3_)_2_, 1e with –CH_2_CH_2_CH_2_COOCH_3_, and 1f with –CH_2_COOCH_3_ at the R^3^ substituent have much lower activity than any other compound with smaller substituent at the same location, like 1a with –CH_2_CH(OH)CH_2_OH, 1c with –CH_2_CH_2_CH_2_OH, and 1d with –CH_2_CH_2_OH.

The CoMFA electrostatic contour map is shown in Figure 4B. The blue (electropositive groups are favorable) and red (electronegative groups are favorable) contours represent 80% and 20% level contributions, respectively. The blue contour around the 2-position of ring C (R^1^ and R^2^ substituents) indicates that substitutions with positively-charged groups are favored for inhibitory activity. This is in agreement with the experimental findings, such as the order of activity for those compounds: 22b (C_9_H_13_N) > 5g (C_6_H_7_N) > 33b (C_9_H_12_O). Compound 1d has a higher activity when compared to 1e, explaining why a large blue contour exists near the 2-position of ring A. In addition, some red contours are observed around ring A, ring B, and ring C, which indicates that electronegative groups would be favorable.

**Figure 5:**
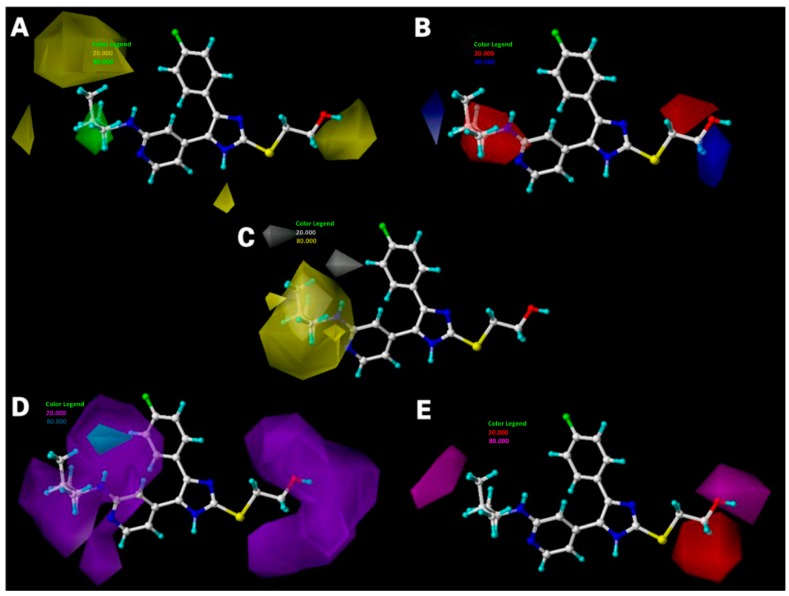
CoMSIA StDev*Coeff (**A**) steric; (**B**) electrostatic; (**C**) hydrophobic; (**D**) H-bond donor and (**E**) H-bond acceptor contour maps. The color code is as follows: (**A**) green and yellow contours indicate favorable and unfavorable bulky groups, respectively; (**B**) blue and red contours indicate favorable and unfavorable electropositive groups, respectively; (**C**) yellow and white contours indicate favorable and unfavorable hydrophobic groups, respectively; (**D**) cyan and purple contours indicate favorable and unfavorable H-bond donor groups, respectively; (**E**) magenta and red contours indicate favorable and unfavorable H-bond acceptor groups, respectively. The compound 3v in ball and stick is displayed as a reference.

The electrostatic contour map of the CoMSIA model is displayed in Figure 5B. The blue (electropositive groups are favorable) and red (electronegative groups are favorable) contours represent 80% and 20% level contributions, respectively. A red region is observed near the 2-position of ring A (R^3^ substituent), suggesting that substitution with negatively-charged group would increase the inhibitory activity. Due to the presence of electronegative groups (–CH_2_CH_2_OH) at the 2-position of ring A, compounds 3v, 3w, and 3u show dramatic increases in activity, which implies that there may exist important hydrogen bond interactions with those groups. In addition, a large red contour near the R^2^ substituent indicates substitution with a negatively-charged group is favored for inhibitory activity. This discovery is well-illustrated by the example that compound 1d has a higher activity than 1c. The location of blue contours is similar to the previous CoMFA model, which is thus not specified here.

The CoMSIA contour map of hydrophobic contribution is described in Figure 5C. In this figure, the yellow (hydrophobic groups are favorable) and white (hydrophobic groups are unfavorable) contours represent 80% and 20% level contributions, respectively. A big yellow contour is observed around the 2-position of ring C (R^1^ substituent), suggesting this region’s taste for hydrophobic substituents. The fact that compounds with high activity, like 22f, 22g, 28l, 35b, and 35d, all have bulky hydrophobic substituents at R^1^ position (occupying the yellow contours) well illustrates this conclusion. Whereas, a medium-sized white contour at the 2-position of ring C (R^2^ substituent) and another two at the left of R^1^ substituent indicate hydrophilic substituents groups (like hydroxy or amido) are favorable for activity. Hence, substitution of bulky hydrophobic moieties with small polar groups on the left extended to the large yellow and medium-sized white regions resulting in a higher inhibitory activity, which can be exemplified by the higher activity of chemical 30 (with C_12_H_16_O_2_), 20 (with C_11_H_14_O_2_), 31 (with C_10_H_12_O_2_), and 5 (with C_9_H_10_O_2_) than their analog compound 18 (with C_11_H_14_O).

The CoMSIA contour map of H-bond donor contribution is described in Figure 5D. In this figure, the cyan (H-bond donor groups are favorable) and purple (H-bond donor groups are unfavorable) contours represent 80% and 20% level contributions, respectively. A small cyan contour map near the R^1^ substituent suggests that the presence of strong electropositive H-bond donors in these regions is favorable for the biological activity. There is a large purple contour map observed around ring C, suggesting its unfavorable effect on the H-bond donor groups. There is another purple contour observed near the R^3^ substituent suggesting an H-bond donor moiety located near these regions will result in impaired biological activity.

The H-bond acceptor contour map of the CoMSIA model is displayed in Figure 5E. The magenta (H-bond acceptor groups are favorable) and red (H-bond acceptor groups are unfavorable) contours represent 80% and 20% level contributions, respectively. A large magenta contour is seen in the vicinity of the OH, which suggests that substitution with a H-bond acceptor group would increase the inhibitory activity. Furthermore, a medium-sized magenta contour map appears near the R^1^ substituent, which is consistent with the findings revealed by the H-bond donor contour map. A relatively larger red contour map near the 2-position of ring A implies that H-bond donor groups could have a positive influence on the inhibitory activity.

### 2.4. Pharmacophore Modeling

**Figure 6:**
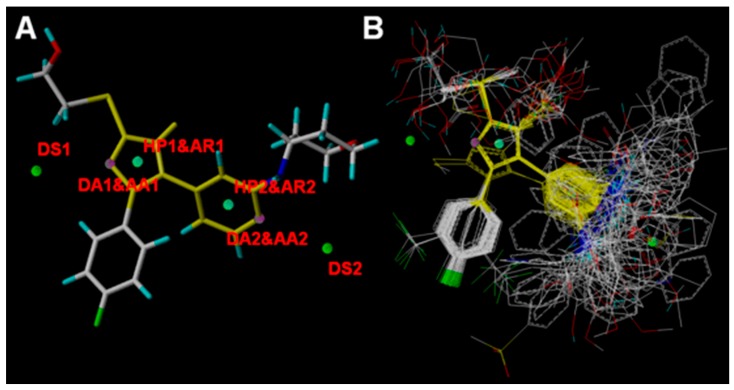
Pharmacophore model of DISCOtech. (**A**) Model_006 with ten features generated presented in template molecule 3v (**B**) Alignment of 100 molecules. AR represents aromatic center; HP refers to hydrophobic center; DA and AA are short for H-bond donor and acceptor atom, respectively; DS and AS refer to H-bond donor site and H-bond acceptor site, respectively. Molecules are colored in yellow and white for common C, blue for N, red for O, cyan for H, and green for F, respectively.

Presently, 100 highly active compounds participated in the establishment of the pharmacophore model in order to identify the features required for TNF-α release inhibitors because of their structural diversity and high activity. The most active compound, 3v, was selected as the reference molecule for the pharmacophore model. The maximum number of conformers generated for each compound was 50, and seven conformers were then selected. In total, eight models were generated using the most active 100 antagonists, and the statistic results of pharmacophore modeling by Distance Comparisons technique (DISCOtech) are listed in Table 2. Ten features were searched for all eight obtained models, and all molecules matched with the obtained models. In general, the DISCOtech pharmacophore model with a relatively high score, more useful features, and moderate pairwise tolerance would be selected as the best model. As depicted in Figure 6A,B, Model_006 was selected as the best model for subsequent studies because of its highest score value (4.1075) and useful features of a higher diversity compared with others.

**Table 2:** Number of models obtained along with the pharmacophoric features and tolerance values for each of the DISCO pharmacophoric.

Model	Size ^a^	Hits ^b^	Score ^c^	Tolerance ^d^	Dmean ^e^
MODEL_006	10	100	4.1075	0.25	4.5615
MODEL_003	10	100	4.1015	0.50	4.5264
MODEL_008	10	100	4.1015	0.50	4.5263
MODEL_007	10	100	3.4537	0.50	3.7971
MODEL_005	10	100	3.4526	0.25	3.7925
MODEL_001	10	100	3.4517	0.25	3.789
MODEL_004	10	100	3.4517	0.25	3.789
MODEL_002	10	100	3.4516	0.25	3.7885

^a^, SIZE, number of features in the model; ^b^, HITS, number of molecules that matched during the research; ^c^, SCORE, an overall measure of fit and of overlap for the entire collection of structure; ^d^, TOLERANCE, initial tolerance setting (from 0.25 to 2.5); ^e^, DMEAN, average inter-point distance.

The DISCOtech model with the alignment of 100 molecules is presented in Figure 6B. All aligned conformers represent stochastic conformations of the molecules and the final alignment shows a satisfactory superimposition of the pharmacophoric points. Figure 6A shows the features arrangement of the optimal pharmacophore model (model_006) obtained with a highest score of 4.1075 and tolerance distance of 0.25 Å. Clearly, the model consists of ten essential features required for high receptor binding affinity, defined as two hydrophobic sites (HP1 and HP2), two aromatic centers (AR1 and AR2), two H-bond donor atoms (DA1 and DA1), two H-bond acceptor atoms (AA1 and AA2), and two H-bond donor sites (DS1 and DS2).

**Figure 7:**
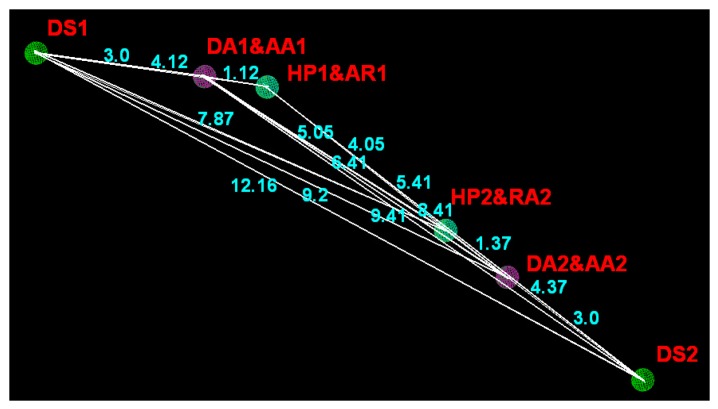
Pharmacophoric features and their distance relation generated by DISCOtech module, AR represents aromatic center; HP refers to hydrophobic center; DA and AA are short for H-bond donor and acceptor atom, respectively; DS and AS refer to H-bond donor site and H-bond acceptor site, respectively.

The pharmacophoric feature points and the distances between pharmacophore features are presented in Figure 7 and Table 3, respectively. The distance between HP1&AR1 in ring A and HP2&AR2 in ring C is 5.05 ± 0.25 Å, and the hydrophobic ring centers or aromatic centers revealed by this model indicate that hydrophobic interaction has important roles in the action mechanism between inhibitors and the TNF-α release inhibitory activity. Moreover, the nitrogen atom at the 3-position of ring A corresponds to the DA1 and AA1 features of the model, with the DS1 representing its counterpart on the putative receptor. Similarly, the nitrogen atom at the 1-position of ring C also corresponds to the DA2 and AA2 features, with the DS2 representing its counterpart on the putative receptor. The distance between those features are: 12.16 ± 0.25 Å (DS1 and DS2), 3.00 ± 0.25 Å (DS1 and DA1&AA1), 9.20 ± 0.25 Å (DS1 and DA2&AA2), 16.41 ± 0.25 Å (DA1&AA1 and DA2&AA2), 9.41 ± 0.25 Å (DA1&AA1 and DS2), 3.00 ± 0.25 Å (DA2&AA2 and DS2). Those important hydrogen bond-donor and hydrogen bond-acceptor features corresponding to H-bond interactions also play an important role in the improvements of the activity of TNF-α release inhibitors. Closer inspection of the pharmacophore model reveals that the hydrogen bond-donor and hydrogen bond-acceptor results are in agreement with the results from the previously reported study results that three important H-bonds were formed with N atom of imidazole (ring A) and 4-pyridyl (ring C): H-bonds (1) between Lys53 and imidazole N3; (2) between the 4-pyridyl N (ring C) and Met109N; and (3) between the 4-pyridyl N (ring C) and Gly110N [26]. All of the features are mapped perfectly for the highly active compounds, whereas the features of inactive compounds are not mapped well. In conclusion, our pharmacophore results reveal that hydrophobic interaction and hydrogen bonds are the crucial factors acting on TNF-α release inhibitory activity, which is also consistent with the 3D-QSAR models.

**Table 3:** Relative intramolecular distances between pharmacophoric feature points for model_006 (Å).

Domain	DS1	DA1/AA1	HP1/AR1	HP2/AR2	AA2/DA2
DA1/AA1	3.00 ± 0.25	–	–	–	–
HP1/AR1	4.12 ± 0.25	1.12 ± 0.25	–	–	–
HP2/AR2	7.87 ± 0.25	5.05 ± 0.25	4.05 ± 0.25	–	–
AA2/DA2	9.20 ± 0.25	6.41 ± 0.25	5.41 ± 0.25	1.37 ± 0.25	–
DS2	12.16 ± 0.25	9.41 ± 0.25	8.41 ± 0.25	4.37 ± 0.25	3.00 ± 0.25

AR represents aromatic center; HP refers to hydrophobic center; DA and AA are short for H-bond donor and acceptor atom, respectively; DS and AS refer to H-bond donor site and H-bond acceptor site, respectively.

## 3. Experimental Section

### 3.1. Dataset and Biological Activity

A total of 151 2-thioimidazoles were used as the data set to derive the 3D-QSAR models in this work, with a wide spectrum of activity values against TNF-α release collected from the work of Stefan A. Laufer and co-workers [2,10,18,19]. The *in vitro* biological activities of these compounds were converted into the corresponding pIC_50_ (−log IC_50_) values, which were used as dependent variables in the *in silico* analyses. All the structures and biological activities of the data set are listed in Table S2. In an approximate ratio of 4:1 the whole data set was divided into training (118) and test (33 molecules) sets by using Kohonen’s self-organizing maps (SOM). In order to classify the dataset, a neural network mapped all the points in a high-dimensional space into a two dimensional space. In this two dimensional Kohonen map, the same neurons or neurons near to each other were occupied by data with similar input.

### 3.2. Molecular Modeling and Alignment Procedure

All molecular modeling and 3D-QSAR studies were performed using the Sybyl 6.9 molecular modeling software package (Tripos Associates, St. Louis, MO, USA). Partial atomic charges were calculated by the Gasteiger-Huckel method [48], while energy minimization and conformational search were performed using Tripos molecular mechanics force field [49] by conjugating method with a convergence criterion of 0.001 kcal/mol. The energy gradient limit was set at 0.05 kcal/mol·Å to obtain the most stable conformation. The predicting ability of the produced models can be done by applying the statistical tests for the continuous QSAR models [50,51].

The structural alignment of the compounds is one of the most important and challenging steps in the development of a successful 3D-QSAR model [52]. Since all the compounds share a common scaffold, it was assumed that each molecule binds into the integrin active site in a similar mode [53]. In the present study, the most potent compound, 3v, was chosen as a template to fit the remaining compounds on the common substructure by using the “align database” command in Sybyl 6.9 (Tripos Associates, St Louis, MO, USA).

### 3.3. CoMFA and CoMSIA

Several approaches have been developed for the 3D-QSAR study and CoMFA and CoMSIA are two of the most popular methods. CoMFA/CoMSIA were anticipated by MTD/Minimal Topological Difference methods [54,55]. These two methods were recently reported in modeling degenerative diseases [56,57,58].

In order to obtain the CoMFA and CoMSIA descriptor fields, a 3D cubic lattice intersection of a regularly-spaced grid of 2.0 Å was generated to encompass the aligned molecules. The grid box dimensions were determined automatically in such a way that region boundaries were extended beyond 4 Å in each direction from coordinates of each molecule. In CoMFA, the steric and electrostatic fields were calculated separately for each molecule using a sp^3^ carbon atom probe with a charge of +1.0 and energy cut-off values of 30 kcal/mol for both steric and electrostatic fields. The probe atom was placed at each lattice point, and the steric and electrostatic interactions with each atom in the molecule were computed using the CoMFA standard scaling.

The same grid was used for the CoMSIA field calculation as that constructed for the CoMFA field calculation. Five descriptors (steric, electrostatic, hydrophobic, hydrogen-bond donor and acceptor) were evaluated using a common probe atom with a radius of 1.0 Å, a +1 charge, hydrophobicity of +1.0, and H-bond donor and acceptor properties of +1.0. The contributions from these descriptors were truncated at 0.3 kcal/mol. Validation of the CoMSIA analysis was performed as described for CoMFA. Due to the different shape of the Gaussian function, CoMSIA similarity indices (*A*_F_) for molecule *j* with atom *i* at grid point *q* are calculated by Equation (1):

(1) $${\text{A}}_{F,K}^{q}\left(j\right)=-\sum^{\text{​}}{\text{ω}}_{\text{probe},\text{k}}{\text{ω}}_{\text{ik}}e^{-\alpha r_{iq}^{2}}$$

where *k* represents the steric, electrostatic, hydrophobic, or H-bond donor or acceptor descriptor. ω_probe,k_ is the probe atom with radius 1.0 Å, charge +1, hydrophobicity +1, H-Bond donating +1, H-bond accepting +1; ω_ik_ is the actual value of the physicochemical property *k* of atom *i*; *r*_iq_ is the mutual distance between the probe atom at grid point *q* and atom *i* of the test molecule. The attenuation factor α was set to 0.3.

### 3.4. Partial Least Square Analysis and Statistical Validation

To quantify the relationship between the structural parameters (CoMFA/CoMSIA interaction energies) and the biological activities, the Partial Least Squares (PLS) algorithm [59,60] was used. The CoMFA/CoMSIA descriptors were used as independent variables, and pIC_50_ values as dependent variables in partial least square regression analysis. PLS is a statistical approach that generalizes and combines features from principal component analysis (PCA) and multiple regressions. It is particularly useful to predict a set of dependent variables from a large set of independent variables when the matrix of predictors has more variables than observations (multicollinearity).

To evaluate the reliability of the models and determine the optimal number of components for further non-cross-validated analysis, cross-validations were carried out using the “leave-one-out” option (LOO), wherein one compound was moved away from the dataset and its activity was predicted by using the model derived from the rest of the dataset. A cross-validated coefficient, *Q*^2^, was subsequently obtained and provided as a statistical index of predictive power. The highest *Q*^2^, corresponding to the smallest predicted error (*SEP*) and the optimal number of component, was used to obtain the final QSAR model. Then, the non-cross-validated models were evaluated by the Pearson coefficient (*R*^2^_ncv_), standard error of estimate (*SEE*), and *F* test ratio. Finally, the CoMFA/CoMSIA results were graphically represented by field contour maps, where the coefficients were generated using the field type “StDev*Coeff”.

In order to evaluate the real predictive ability of the best models generated by the CoMFA/CoMSIA analyses using the same training set, the pIC_50_ values of test compounds are treated as the external validation set. The predictive ability of the model was evaluated by defining *R*^2^_pre_, which was then obtained with the following formula:

(2) $${R^{2}}_{\text{pre}}=\sqrt{\left(SD-PRESS\right)/SD}$$

where *SD* denotes the sum of squared deviation between the biological activities of the test set molecule and the mean activity of the training set molecules, *PRESS* represents the sum of squared deviations between the experimental and predicted activities of the test molecules.

### 3.5. DISCOtech Analysis

To identify the general pharmacophoric features the dataset was studied, at first, by the Distance Comparisons technique, a distance constraint pharmacophore building method. DISCOtech, a well-established module for designing pharmacophoric maps and frequently used in the process of virtual screening to discover new leads, is an enhanced, faster version of DISCO (DIStance Comparison) [61]. DISCOtech identifies features that could be elements in a pharmacophore model from a set of molecules that are related by their ability to bind to a common binding site [62]. DISCOtech, operating in distance space, can carry out clique detection to generate pharmacophore hypotheses for up to 300 conformers per molecule [63,64,65]. Elements considered in developing the pharmacophore model include hydrogen bond donor atoms, hydrogen bond acceptor atoms, hydrogen donor and acceptor site, charge centers, centers of mass of hydrophobic rings, aromatic rings, and positive NA (numerical aperture) stochastic search method was run to generate a maximum of 50 conformers for each molecule on the basis of maximum diversity to cover as many probable conformers as possible, and seven conformers were then selected. Min 4 and Max 16 features were allowed to be found during the analysis. Tanimoto threshold was set as 0.85. All other parameters were retained as default values.

## 4. Conclusions

**Figure 8:**
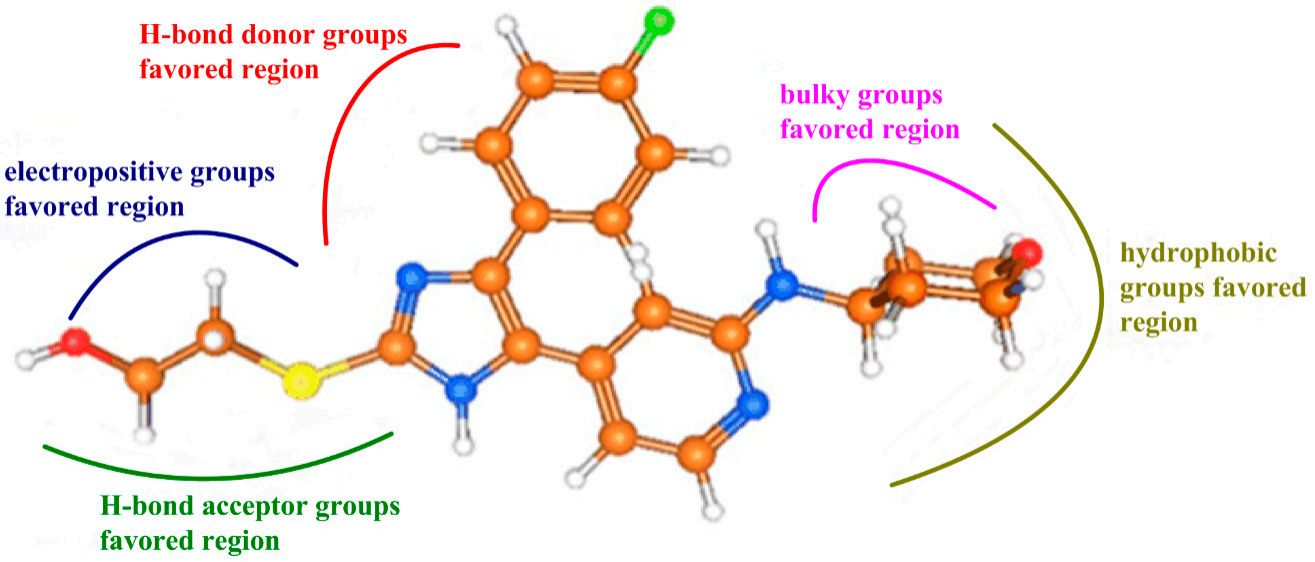
The interaction features of compound 3v impacting the antagonistic activity obtained from our present work.

In the present work, a series of CoMFA, CoMSIA, and pharmacophore studies were performed on 151 imidazole-based TNF-α release inhibitors (see Figure 8). Both the reliability and predictivity of the resultant optimal CoMFA and CoMSIA models (with *Q*^2^ = 0.557, *R*^2^_ncv_ = 0.740, *R*^2^_pre_ = 0.748 and *Q*^2^ = 0.598, *R*^2^_ncv_ = 0.767, *R*^2^_pre_ = 0.860, respectively) were validated by their high *Q*^2^, *R*^2^_ncv_, and *R*^2^_pre_ values. The corresponding contour maps generated by these models provide more diverse information about the key intermolecular interactions of inhibitors with the surrounding environment. A good consistency was also observed between the QSAR models and pharmacophore modeling studies. To sum up, our findings are:

1. Bulky substituents at R^1^ position may improve the inhibitory activity.
2. Hydrophobic groups around R^1^ substituent are helpful to enhance the potency of the inhibitors.
3. Electropositive groups at R^3^ position are beneficial to improve the biological activity of inhibitors.
4. H-bond donor groups around ring C and acceptor groups at R^3^ substituent promote the inhibitory activity, respectively.
5. Hydrophobic interaction and hydrogen bonds were the crucial factors acting on the inhibitory activity of TNF-α release.

We do hope the results of the present study may provide further support to the design of imidazoles as potential inhibitors of TNF-α release.

## Supplementary Materials

Supplementary materials can be accessed at: http://www.mdpi.com/1422-0067/16/09/20118/s1.
