# Supplementary material for: Insight into the Structural Determinants of Imidazole Scaffold-Based Derivatives as TNF-α Release Inhibitors by in Silico Explorations
Source: Int J Mol Sci. 2015 Aug 25;16(9):20118–38. doi: 10.3390/ijms160920118 (PMC4613192; doi:10.3390/ijms160920118)
Supplement: Supplementary file 1 [file ijms-16-20118-s001.pdf]

Supporting Information

Table S1. Experimental and predicted activities (pIC_{50}) for the training and test set compounds employed in CoMFA and CoMSIA analyses. The letter after the number represents different molecular structure.

Compound	Actual	CoMFA		CoMSIA	
		Predicted	Residuals	Predicted	Residuals
**Training Set**					
1	4.612254	4.331	0.281254	4.505	0.107254
10	5.200659	5.365	−0.16434	5.048	0.152659
13	4.513711	4.477	0.036711	4.377	0.136711
14	5.236722	4.914	0.322722	5.232	0.004722
16	4.658961	4.500	0.158961	4.551	0.107961
17	4.669992	4.966	−0.29601	4.763	−0.09301
1A	5.471083	5.361	0.110083	5.548	−0.07692
1B	4.595508	4.178	0.417508	4.183	0.412508
1D	5.375718	5.074	0.301718	5.086	0.289718
1E	4.495937	4.633	−0.13706	4.573	−0.07706
1F	4.036543	4.528	−0.49146	4.289	−0.25246
2	4.676954	5.164	−0.48705	5.127	−0.45005
20	4.424812	4.903	−0.47819	4.715	−0.29019
22	5.436519	5.124	0.312519	4.98	0.456519
22B	5.363512	5.283	0.080512	5.193	0.170512
22C	6.172631	5.132	1.040631	5.622	0.550631
22D	5.573489	5.828	−0.25451	5.767	−0.19351
22E	5.551294	5.647	−0.09571	5.628	−0.07671
22F	5.405607	4.967	0.438607	5.146	0.259607
22G	6.455932	6.073	0.382932	6.145	0.310932
22H	4.361511	4.433	−0.07149	4.556	−0.19449
22I	4.360514	5.129	−0.76849	4.928	−0.56749
22J	4.279014	4.003	0.276014	4.44	−0.16099
22K	4.653647	4.701	−0.04735	5.217	−0.56335
22L	4.288193	4.687	−0.39881	4.783	−0.49481
22M	4.552842	4.782	−0.22916	4.832	−0.27916
23	5.189096	5.203	−0.0139	5.015	0.174096
24	4.26552	4.329	−0.06348	4.443	−0.17748
25	4.573977	4.826	−0.25202	4.786	−0.21202
25A	4.419075	4.353	0.066075	4.607	−0.18792
25C	4.673664	5.209	−0.53534	5.239	−0.56534
25D	4.638272	4.55	0.088272	4.733	−0.09473
25E	5.333482	4.698	0.635482	4.912	0.421482
26	4.300596	4.385	−0.0844	4.578	−0.2774
26A	5.31158	5.176	0.13558	5.209	0.10258
26B	4.838632	5.324	−0.48537	5.255	−0.41637
26C	4.255707	4.407	−0.15129	4.528	−0.27229
26D	5.258061	5.232	0.026061	5.443	-0.18494
26E	5.129011	5.115	0.014011	5.159	−0.02999
26F	5.364516	5.189	0.175516	5.387	−0.02248

Table S1. *Cont.*

Compound	Actual	CoMFA		CoMSIA	
		Predicted	Residuals	Predicted	Residuals
26G	5.006564	5.225	−0.21844	5.366	−0.35944
27	4.493089	4.532	−0.03891	4.699	−0.20591
27A	4.571865	4.723	−0.15113	4.778	−0.20613
28	4.21674	4.230	−0.01326	4.279	−0.06226
28A	5.226214	5.525	−0.29879	5.131	0.095214
28B	5.481486	5.364	0.117486	5.194	0.287486
28C	4.559091	5.485	−0.92591	5.187	−0.62791
28D	6.441291	5.702	0.739291	5.613	0.828291
28F	5.434152	5.520	−0.08585	5.520	−0.08585
28G	4.801343	5.486	−0.68466	5.460	−0.65866
28I	6.20621	5.487	0.71921	5.488	0.71821
28J	5.614394	5.476	0.138394	5.467	0.147394
28L	6.777284	6.238	0.539284	6.291	0.486284
28M	6.361511	6.114	0.247511	6.290	0.071511
28O	5.399027	5.378	0.021027	5.337	0.062027
29	4.500588	4.465	0.035588	4.498	0.002588
29A	5.004804	4.678	0.326804	4.575	0.429804
29B	5.533132	5.443	0.090132	5.395	0.138132
3	5.092642	4.948	0.144642	4.911	0.181642
30A	5.69037	6.192	−0.50163	6.076	−0.38563
30B	6.380907	6.039	0.341907	6.081	0.299907
30D	6.357535	6.311	0.046535	6.149	0.208535
30E	6.621602	6.378	0.243602	6.023	0.598602
30G	6.167491	6.292	−0.12451	5.854	0.313491
30H	6.696804	6.372	0.324804	6.542	0.154804
31	4.603104	4.905	−0.3019	4.804	−0.2009
31A	5.284833	5.793	−0.50817	5.414	−0.12917
32	4.26019	4.422	−0.16181	4.377	−0.11681
33	4.9914	4.726	0.2654	4.742	0.2494
33B	4.35164	4.811	−0.45936	4.783	−0.43136
33C	4.737549	4.399	0.338549	4.451	0.286549
33E	4.950782	5.225	−0.27422	5.072	−0.12122
33G	4.369572	4.491	−0.12143	4.426	−0.05643
34	4.43086	4.427	0.00386	4.73	−0.29914
34B	4.617983	5.337	−0.71902	4.823	−0.20502
35	4.512862	4.614	−0.10114	4.73	−0.21714
35A	4.69897	5.207	−0.50803	5.034	−0.33503
35B	5.767004	5.329	0.438004	5.13	0.637004
35C	5.006123	5.251	−0.24488	5.156	−0.14988
35D	6.236572	5.366	0.870572	5.236	1.000572
35F	4.420216	4.419	0.001216	4.446	−0.02578
35G	5.447332	4.759	0.688332	4.921	0.526332
35I	5.230623	5.455	−0.22438	4.964	0.266623
35J	5.782516	5.574	0.208516	5.439	0.343516
35O	5.195179	5.435	−0.23982	5.005	0.190179

Table S1. *Cont.*

Compound	Actual	CoMFA		CoMSIA	
		Predicted	Residuals	Predicted	Residuals
39	4.687188	4.932	−0.24481	4.774	−0.08681
3B	5.573489	5.318	0.255489	5.346	0.227489
3C	6.774691	6.607	0.167691	6.95	−0.17531
3E	5.242300	6.136	−0.8937	6.454	−1.211700
3F	6.737549	6.879	−0.14145	6.698	0.039549
3H	6.899629	6.570	0.329629	6.619	0.280629
3I	6.882729	6.974	−0.09127	6.94	−0.05727
3K	7.431798	6.91	0.521798	7.234	0.197798
3L	6.777284	6.963	−0.18572	7.156	−0.37872
3N	6.744727	6.703	0.041727	6.943	−0.19827
3O	6.910095	6.634	0.276095	6.665	0.245095
3P	4.34218	4.292	0.05018	4.141	0.20118
3Q	4.182435	4.551	−0.36857	4.159	0.023435
3R	4.712198	5.056	−0.3438	4.47	0.242198
3S	5.048662	5.696	−0.64734	5.054	−0.00534
3T	4.893809	5.449	−0.55519	5.444	−0.55019
3U	6.88941	6.317	0.57241	6.611	0.27841
3V	7.09691	6.806	0.29091	7.275	−0.17809
3W	6.8041	6.888	−0.0839	7.139	−0.3349
40	4.600672	4.551	0.049672	4.553	0.047672
42	4.569925	4.504	0.065925	4.504	0.065925
43	4.569925	4.505	0.064925	4.544	0.025925
45	4.609418	4.805	−0.19558	4.924	−0.31458
47	4.650722	4.464	0.186722	4.623	0.027722
5	4.840132	4.839	0.001132	4.818	0.022132
5A	4.524329	4.39	0.134329	4.614	−0.08967
5F	4.293282	4.309	−0.01572	4.178	0.115282
5G	5.062984	4.612	0.450984	4.643	0.419984
5I	4.354578	3.979	0.375578	4.202	0.152578
5J	4.145694	3.745	0.400694	4.083	0.062694
6	4.630042	4.745	−0.11496	4.832	−0.20196
7	4.744486	5.079	−0.33451	4.917	−0.17251
9	5.003051	4.798	0.205051	4.812	0.191051
**Test Set**					
11	4.432151	4.473	−0.04085	4.86	−0.42785
15	4.688034	4.69	−0.00197	4.645	0.043034
18	4.233067	4.314	−0.08093	4.417	−0.18393
1C	5.174574	5.103	0.071574	5.354	−0.17943
21	5.247952	5.01	0.237952	4.92	0.327952
25B	4.91364	4.842	0.07164	4.754	0.15964
26H	5.20621	5.275	−0.06879	5.498	−0.29179
27B	4.829738	4.956	−0.12626	4.991	−0.16126
28E	6.339135	5.88	0.459135	6.171	0.168135
28H	5.267606	5.52	−0.25239	5.52	−0.25239
28K	5.342944	5.38	−0.03706	5.357	−0.01406

Table S1. *Cont.*

Compound	Actual	CoMFA		CoMSIA	
		Predicted	Residuals	Predicted	Residuals
28N	6.154902	5.334	0.820902	5.793	0.361902
30	4.807433	4.544	0.263433	4.665	0.142433
30C	6.288193	6.078	0.210193	6.108	0.180193
30F	5.974694	6.274	−0.29931	6.194	−0.21931
30I	6.413413	6.586	−0.17259	6.364	0.049413
31B	5.774691	5.981	−0.20631	5.756	0.018691
33A	4.349692	4.543	−0.19331	4.619	−0.26931
33D	4.415669	4.647	−0.23133	4.629	−0.21333
35H	4.939302	4.919	0.020302	5	−0.0607
35L	4.759451	5.459	−0.69955	4.97	−0.21055
3A	6.028724	5.959	0.069724	5.577	0.451724
3D	6.109579	6.501	−0.39142	6.635	−0.52542
3G	6.806875	6.665	0.141875	6.544	0.262875
3J	6.838632	6.563	0.275632	6.642	0.196632
3M	6.856985	6.948	−0.09101	6.801	0.055985
41	4.349984	4.319	0.030984	4.444	−0.09402
44	4.704433	4.593	0.111433	4.585	0.119433
48	4.748119	4.363	0.385119	4.554	0.194119
5E	4.443697	4.361	0.082697	4.296	0.147697
5H	4.774691	4.744	0.030691	4.846	−0.07131
5K	4.077794	3.8	0.277794	3.979	0.098794
8	4.741842	4.713	0.028842	4.751	−0.00916

Table S2. Representative skeletons and molecular structures of 3- and 4-substituted 2-thioimidazoles and their TNF-α IC_{50} values. * Molecules belonged to the test set. The letter after the number represents different molecular structure.

Number	Compound	R^1	R^2	R^3	R^4	IC_{50} (μM)
				Scaffold A (SA) 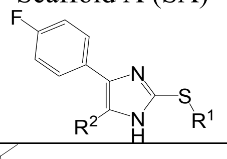		
1	5a	$-CH_3$	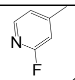	–	–	29.90
2	5g	$-CH_3$	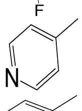	–	–	8.65
3	5h *	$-CH_3$	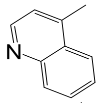	–	–	16.80
4	5e *	$-C_2H_5$	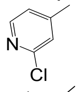	–	–	36.00
5	5f	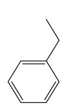	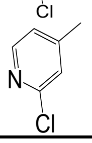	–	–	50.90

Table S2. *Cont.*

Number	Compound	R¹	R²	R³	R⁴	IC_{50} (μM)
				Scaffold B (SB) 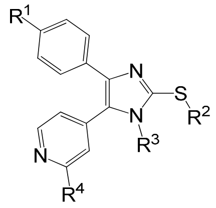		
6	1	–F	$–CH_3$	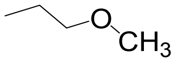	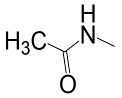	24.42
7	10	–F	$–CH_3$	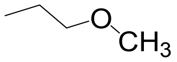	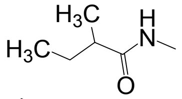	6.30
8	11 *	–F	$–CH_3$	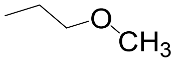	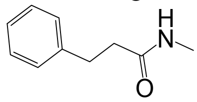	36.97
9	13	–F	$–CH_3$	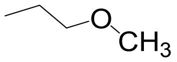	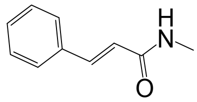	30.64
10	14	–F	$–CH_3$	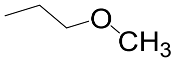	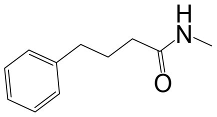	5.80
11	15 *	–F	$–CH_3$	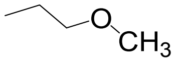	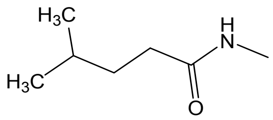	20.51
12	16	–F	$–CH_3$	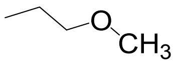	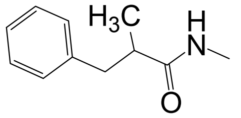	21.93
13	17	–F	$–CH_3$	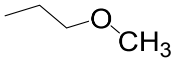	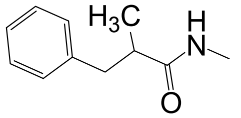	21.38
14	18 *	–F	$–CH_3$	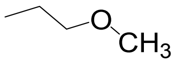	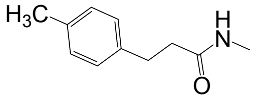	58.47
15	1a	–F	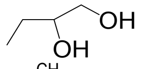	–H	–H	3.38
16	1b	–F	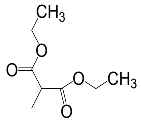	–H	–H	25.38
17	1c *	–F	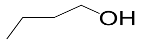	–H	–H	6.69
18	1d	–F	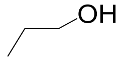	–H	–H	4.21
19	1e	–F	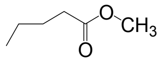	–H	–H	31.92
20	1f	–F	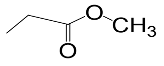	–H	–H	91.93
21	2	–F	$–CH_3$	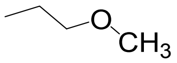	$–NH_2$	21.04
22	20	–F	$–CH_3$	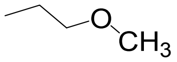	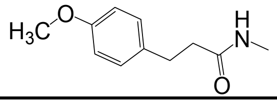	37.6

Table S2. *Cont.*

Number	Compound	R^1	R^2	R^3	R^4	IC_{50} (μM)
23	21 *	–F	$–CH_3$	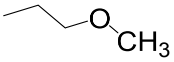	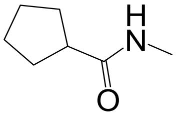	5.65
24	22	–F	$–CH_3$	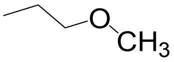	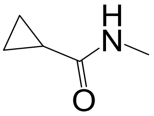	3.66
25	22b	–F	$–CH_3$	–H	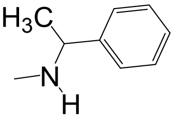	4.33
26	22c	–F	$–CH_3$	–H	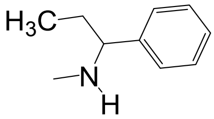	0.67
27	22d	–F	$–CH_3$	–H	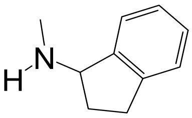	2.67
28	22e	–F	$–CH_3$	–H	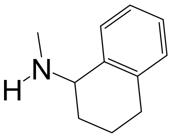	2.81
29	22f	–F	$–CH_3$	–H	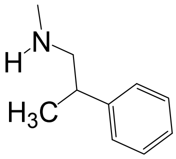	3.93
30	22g	–F	$–CH_3$	–H	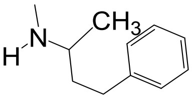	0.35
31	22h	–F	$–CH_3$	–H	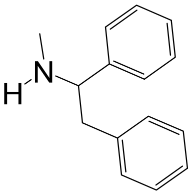	43.5
32	22i	–F	$–CH_3$	–H	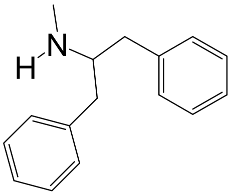	43.6
33	22j	–F	$–CH_3$	–H	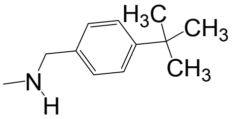	52.6
34	22k	–F	$–CH_3$	–H	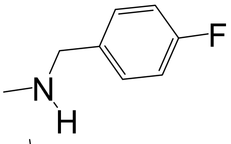	22.2
35	22l	–F	$–CH_3$	–H	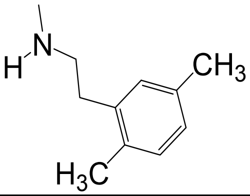	51.5

Table S2. *Cont.*

Number	Compound	R^1	R^2	R^3	R^4	IC_{50} (μM)
36	22m	–F	$–CH_3$	–H	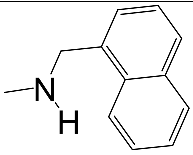	28
37	23	–F	$–CH_3$	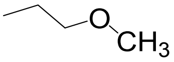	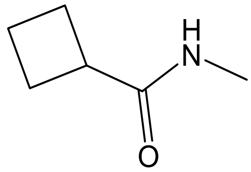	6.47
38	24	–F	$–CH_3$	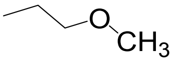	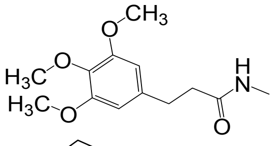	54.26
39	25	–F	$–CH_3$	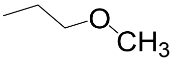	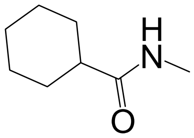	26.67
40	26	–F	$–CH_3$	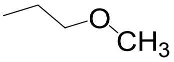	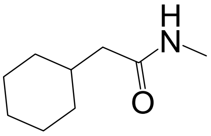	50.05
41	26a	–F	$–CH_3$	–H	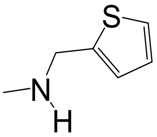	4.88
42	26b	–F	$–CH_3$	–H	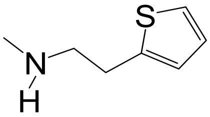	14.50
43	26c	–F	$–CH_3$	–H	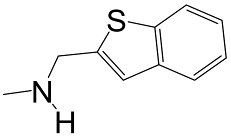	55.50
44	26d	–F	$–CH_3$	–H	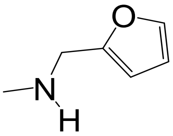	5.52
45	26e	–F	$–CH_3$	–H	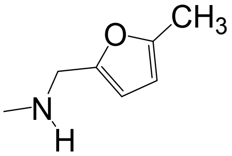	7.43
46	26f	–F	$–CH_3$	–H	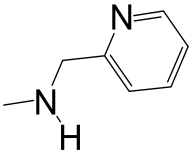	4.32
47	26g	–F	$–CH_3$	–H	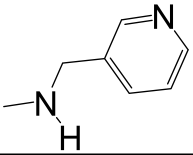	9.85

Table S2. *Cont.*

Number	Compound	R¹	R²	R³	R⁴	IC_{50} (μM)
48	26h *	–F	–CH_3	–H	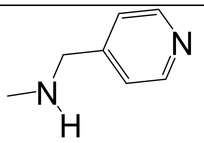	6.22
49	27	–F	–CH_3	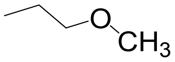	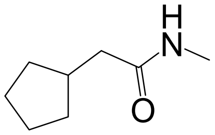	32.13
50	28	–F	–CH_3	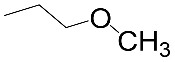	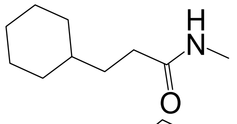	60.71
51	28a	–F	–CH_3	–H	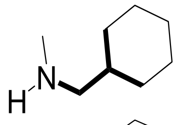	5.94
52	28b	–F	–CH_3	–H	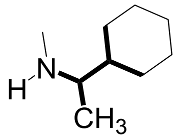	3.30
53	28c	–F	–CH_3	–H	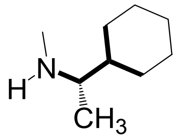	27.60
54	28d	–F	–CH_3	–H	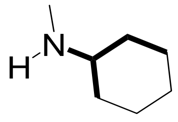	0.36
55	28e *	–F	–CH_3	–H	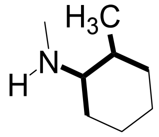	0.458
56	28f	–F	–CH_3	–H	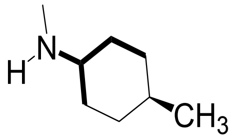	3.68
57	28g	–F	–CH_3	–H	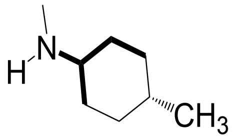	15.80
58	28h *	–F	–CH_3	–H	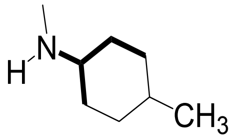	5.40
59	28i	–F	–CH_3	–H	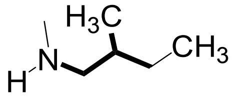	0.62
60	28j	–F	–CH_3	–H	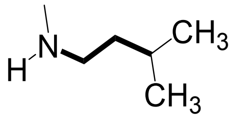	2.43
61	28k *	–F	–CH_3	–H	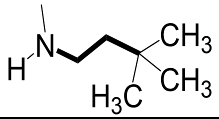	4.54

Table S2. *Cont.*

Number	Compound	R[1]	R[2]	R[3]	R[4]	IC_{50} (μM)
62	28l	–F	$–CH_3$	–H	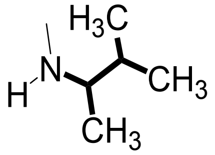	0.17
63	28m	–F	$–CH_3$	–H	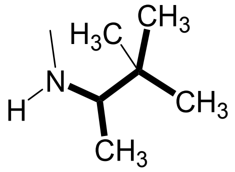	0.44
64	28n *	–F	$–CH_3$	–H	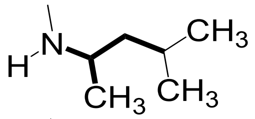	0.70
65	28o	–F	$–CH_3$	–H	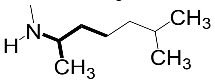	3.99
66	29	–F	$–CH_3$	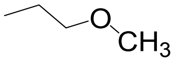	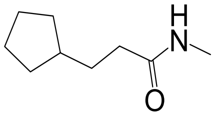	31.58
67	3	–F	$–CH_3$	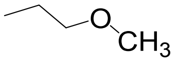	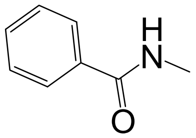	8.08
68	30 *	–F	$–CH_3$	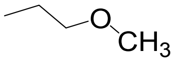	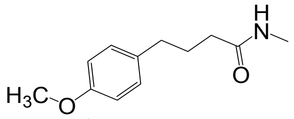	15.58
69	30a	–F	$–CH_3$	–H	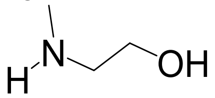	2.04
70	30b	–F	$–CH_3$	–H	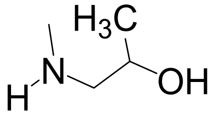	0.42
71	30c *	–F	$–CH_3$	–H	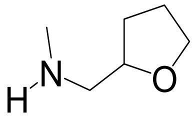	0.52
72	30d	–F	$–CH_3$	–H	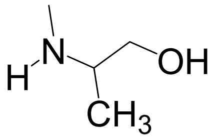	0.44
73	30e	–F	$–CH_3$	–H	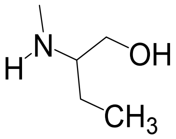	0.24
74	30f *	–F	$–CH_3$	–H	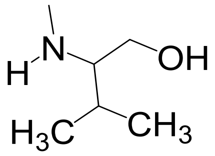	1.06
75	30g	–F	$–CH_3$	–H	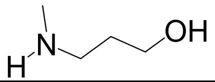	0.68

Table S2. *Cont.*

Number	Compound	R[1]	R[2]	R[3]	R[4]	IC50 (μM)
76	30h	–F	$–CH_3$	–H	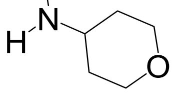	0.20
77	30i *	-F	$–CH_3$	–H	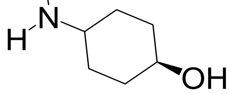	0.39
78	31	–F	$–CH_3$	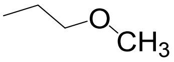	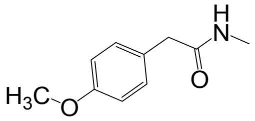	24.94
79	32	–F	$–CH_3$	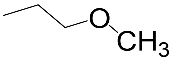	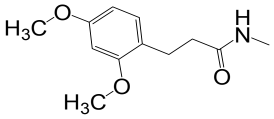	54.93
80	33	–F	$–CH_3$	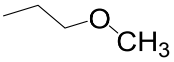	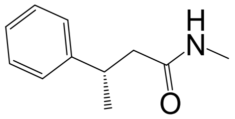	10.20
81	33a *	–F	$–CH_3$	–H	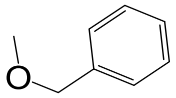	44.70
82	33b	–F	$–CH_3$	–H	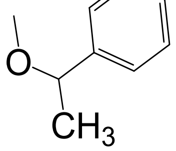	44.50
83	33c	–F	$–CH_3$	–H	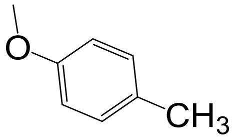	18.30
84	33d *	–F	$–CH_3$	–H	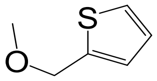	38.40
85	33e	–F	$–CH_3$	–H	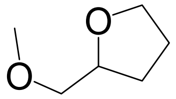	11.20
86	33g	–F	$–CH_3$	–H	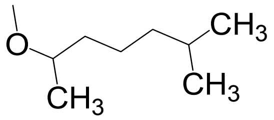	42.70
87	34	–F	$–CH_3$	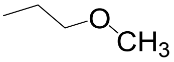	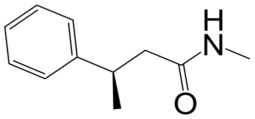	37.08
88	34b	–F	$–CH_3$	–H	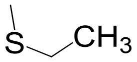	24.10
89	35	–F	$–CH_3$	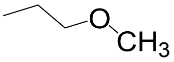	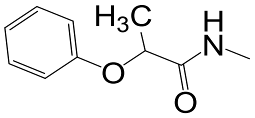	30.70
90	35a	–F	$–CH_3$	$–CH_3$	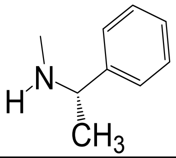	20.00

Table S2. *Cont.*

Number	Compound	R^1	R^2	R^3	R^4	IC$_{50}$ (μM)
91	35b	–F	–CH_3	–CH_3	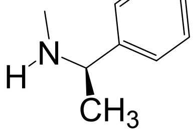	1.71
92	35c	–F	–CH_3	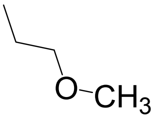	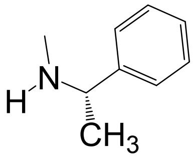	9.86
93	35d	–F	–CH_3	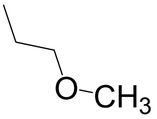	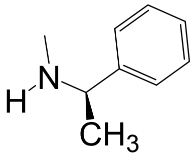	0.58
94	35f	–F	–CH_3	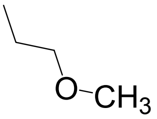	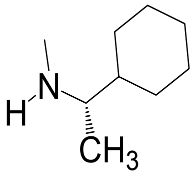	38. 00
95	35g	–F	–CH_3	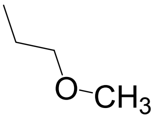	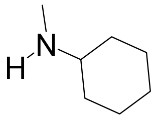	3.57
96	35h *	–F	–CH_3	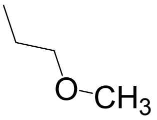	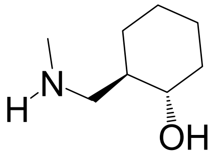	11.5
97	35i	–F	–CH_3	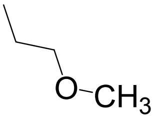	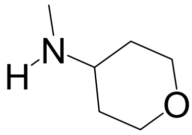	5.88
98	35j	–F	–CH_3	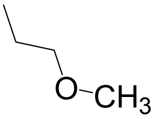	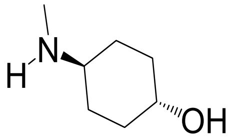	1.65
99	35l *	–F	–CH_3	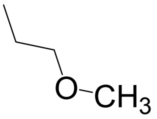	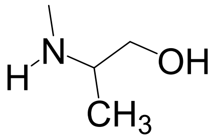	17.40
100	35o	–F	–CH_3	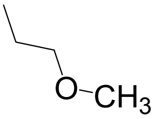	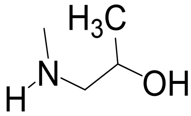	6.38
101	39	–F	–CH_3	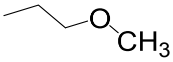	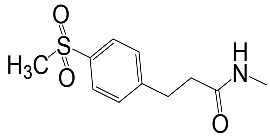	20.55
102	3a *	–F	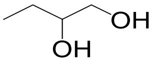	–H	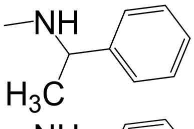	0.94
103	3b	–F	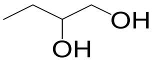	–H	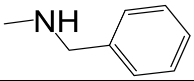	2.67

Table S2. *Cont.*

Number	Compound	R[1]	R[2]	R[3]	R[4]	IC_{50} (μM)
104	3c	–F	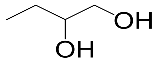	–H	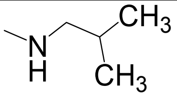	0.17
105	3d *	–F	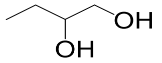	–H	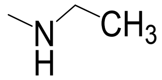	0.78
106	3e	–F	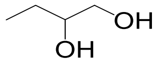	–H	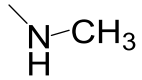	5.72
107	3f	–F	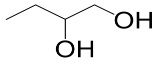	–H	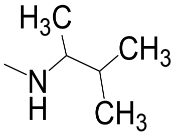	0.18
108	3g *	–F	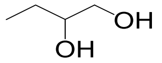	–H	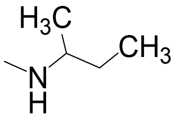	0.16
109	3h	–F	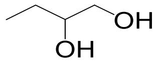	–H	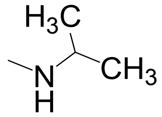	0.13
110	3i	–F	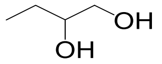	–H	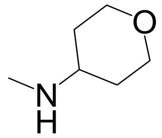	0.13
111	3j *	–F	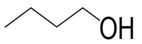	–H	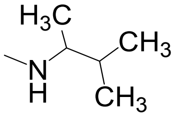	0.15
112	3k	–F	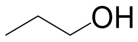	–H	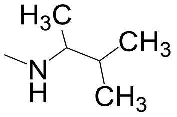	0.04
113	3l	–F	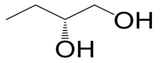	–H	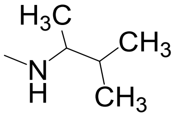	0.17
114	3m *	–F	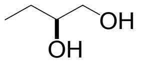	–H	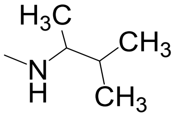	0.14
115	3n	–F	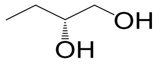	–H	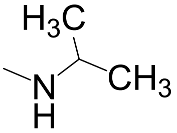	0.18
116	3o	–F	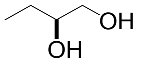	–H	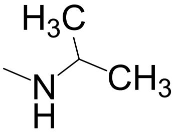	0.12
117	3p	–F	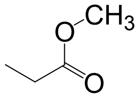	–H	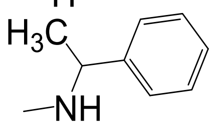	45.48
118	3q	–F	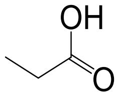	–H	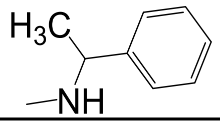	65.70

Table S2. *Cont.*

Number	Compound	R^1	R^2	R^3	R^4	IC_{50} (μM)
119	3r	–F	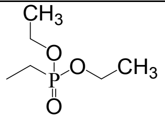	–H	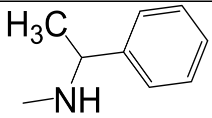	19.40
120	3s	–F	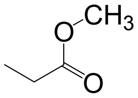	–H	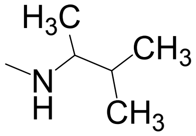	8.94
121	3t	–F	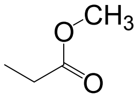	–H	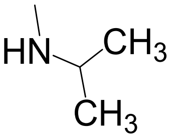	12.77
122	3u	–F	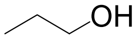	–H	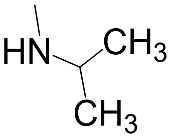	0.13
123	3v	–F	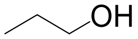	–H	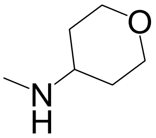	0.08
124	3w	–F	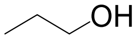	–H	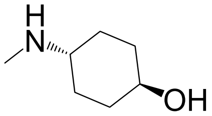	0.16
125	40	–F	–CH_3	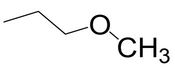	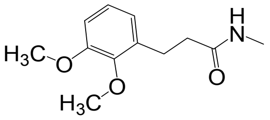	25.08
126	41 *	–F	–CH_3	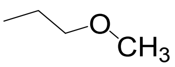	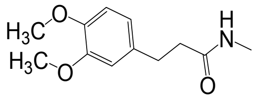	44.67
127	42	–F	–CH_3	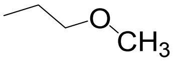	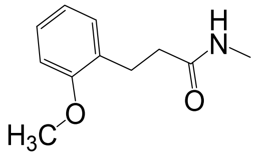	26.92
128	43	–F	–CH_3	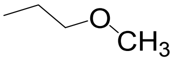	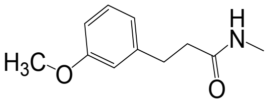	26.92
129	44 *	–F	–CH_3	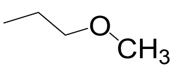	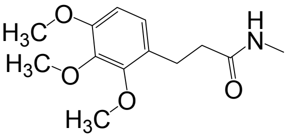	19.75
130	45	–F	–CH_3	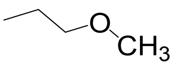	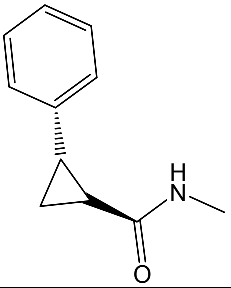	24.58

Table S2. *Cont.*

Number	Compound	R^1	R^2	R^3	R^4	IC$_{50}$ (µM)
131	47	–F	–CH$_3$	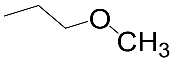	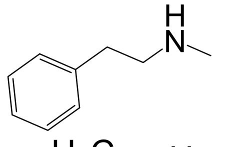	22.35
132	48 *	–F	–CH$_3$	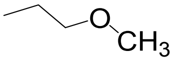	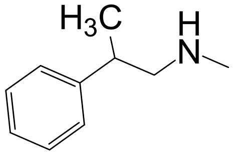	17.86
133	5	–F	–CH$_3$	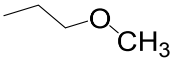	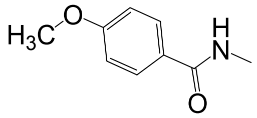	14.45
134	6	–F	–CH$_3$	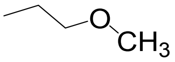	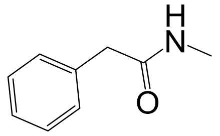	23.44
135	7	–F	–CH$_3$	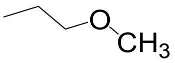	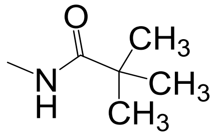	18.01
136	8 *	–F	–CH$_3$	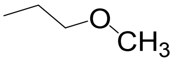	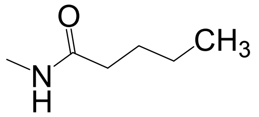	18.12
137	9	–F	–CH$_3$	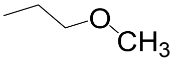	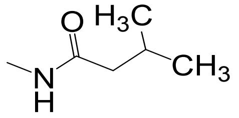	9.93

Scaffold C (SC)

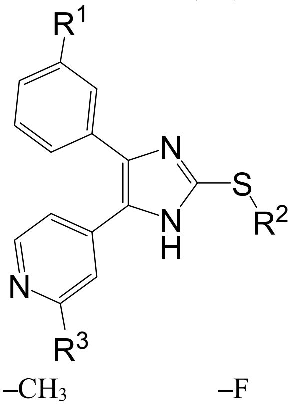

Number	Compound	R^1	R^2	R^3	R^4	IC$_{50}$ (µM)
138	5i	–CF$_3$	–CH$_3$	–F	–	44.2
139	5j	–CF$_3$	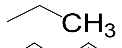	–F	–	71.5
140	5k *	–CF$_3$	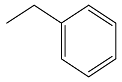	–F	–	83.60
141	25a	–CF$_3$	–CH$_3$	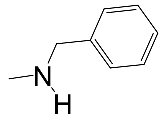	–	38.10
142	25b *	–CF$_3$	–CH$_3$	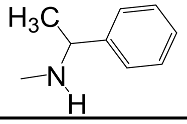	–	12.20

Table S2. *Cont.*

Number	Compound	R^1	R^2	R^3	R^4	IC_{50} (μM)
143	25c	$–CF_3$	$–CH_3$	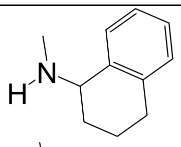	–	21.20
144	25d	$–CF_3$	$–CH_3$	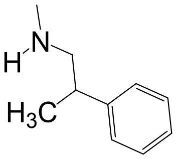	–	23.00
145	25e	$–CF_3$	$–CH_3$	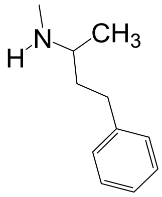	–	4.64
146	27a	$–CF_3$	$–CH_3$	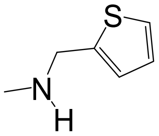	–	26.80
147	27b *	$–CF_3$	$–CH_3$	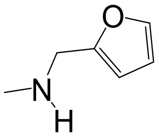	–	14.8
148	29a	$–CF_3$	$–CH_3$	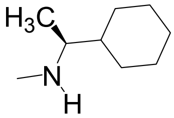	–	9.89
149	29b	$–CF_3$	$–CH_3$	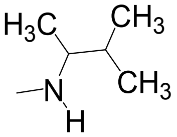	–	2.93
150	31a	$–CF_3$	$–CH_3$	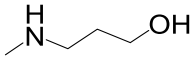	–	5.19
151	31b *	$–CF_3$	$–CH_3$	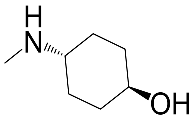	–	1.68
